# Epilepsia Open—September 2022—Announcements

**DOI:** 10.1002/epi4.12639

**Published:** 2022-09-01

## ILAE Congresses

### 11th Summer School for Neuropathology and Epilepsy Surgery (INES 2022)

8‐11 September 2022

Erlangen, Germany

https://www.ilae.org/congresses/11th‐international‐summer‐school‐for‐neuropathology‐and‐epilepsy‐surgery‐ines‐2021

### XII Congreso Latinoamericano de Epilepsia (LAEC 2022)

1‐4 October 2022

Medellín, Colombia

www.ilae.org/laec2022

### 14th Asian & Oceanian Epilepsy Congress

17‐19 November 2022

Online

www.ilae.org/aoec2022

### 35th International Epilepsy Congress

2‐6 September 2023

Dublin, Ireland

www.ilae.org/iec2023

## OTHER CONGRESSES

### 9th International Course on Drug Resistant Epilepsies

4‐10 September 2022

Tagliacozzo, Italy

https://www.ilae.org/congresses/9th‐international‐course‐on‐drug‐resistant‐epilepsies

### 14th International Epilepsy Colloquium

14‐16 September 2022

Lausanne, Switzerland

https://www.ilae.org/congresses/14th‐international‐epilepsy‐colloquium

### Irish Epilepsy League Annual Meeting

16 September 2022

Dublin, Ireland

https://www.ilae.org/congresses/irish‐epilepsy‐league‐annual‐meeting1

### 8th London‐Innsbruck Colloquium on Status Epilepticus and Acute Seizures

17‐20 September 2022

Salzburg, Austria

https://www.ilae.org/congresses/8th‐london‐innsbruck‐colloquium‐on‐status‐epilepticus‐and‐acute‐seizures

### 15th Baltic Sea Summer School on Epilepsy

21‐29 September 2022

Online

https://www.ilae.org/congresses/15th‐baltic‐sea‐summer‐school‐on‐epilepsy

### Canadian League Against Epilepsy 2022 Scientific Meeting

23‐25 September 2022

Kelowna, BC, Canada

https://www.ilae.org/congresses/canadian‐league‐against‐epilepsy‐2022‐scientific‐meeting

### 5th Swiss Federation of Clinical Neuro‐Societies Congress

28‐30 September 2022

Basel, Switzerland

https://sfcns2022.congress‐imk.ch/frontend/index.php

### Cleveland Clinic Epilepsy Update and Review Course

28‐30 September 2022

Ohio, USA & Online

https://www.clevelandclinicmeded.com/live/courses/EpilepsyUpdate22/

### 24èmes Journées Françaises de l’Épilepsie

4‐7 October 2022

Grenoble, France

https://www.ilae.org/congresses/24‐mes‐journ‐es‐fran‐aises‐de‐l‐pilepsie

### 10th Migration and Caucasian Course on Epileptology

9‐12 October 2022

Batumi, Georgia

https://www.ilae.org/congresses/10th‐migration‐and‐caucasian‐course‐on‐epileptology

### 4th Bologna EPIPED‐EEG Course: EEG Interpretation in Pediatric Epilepsies

9‐13 October 2022

Bologna, Italy

http://www.epiped‐eeg‐course.com/

### 2022 ILAE British Branch Annual Scientific Meeting

12‐14 October 2022

Cardiff, United Kingdom

https://www.ilaebritishconference.org.uk/

### Shiraz Annual Epilepsy (Semiology‐EEG) Course

12‐14 October 2022

Shiraz, Iran

https://www.ilae.org/congresses/shiraz‐annual‐epilepsy‐semiology‐eeg‐course

### Imaging Avanzato in Epilessia: Corso Pratico Residenziale LICE

21‐24 October 2022

Bologna, Italy

https://www.ilae.org/congresses/imaging‐avanzato‐in‐epilessia

### Epilepsy Society of Australia 36th Annual Scientific Meeting

26‐28 October 2022

Adelaide, Australia

https://www.ivvy.com.au/event/ESA2022/

### 4th International Video‐EEG Course in Pediatric Epilepsies: From seizures to Syndromes

27‐29 October 2022

Madrid, Spain

https://2022.videoeeg.es/

### ILAE British Branch 2022 Epilepsy Neuroimaging Course

29‐31 October 2022

Chalfont, United Kingdom & Online

https://ilaebritish.org.uk/events/4th‐ilae‐british‐branch‐epilepsy‐neuroimaging‐course/

### ILAE British Branch Clinical Epilepsy 1‐Day Course for Junior Doctors

12 November 2022

Birmingham, United Kingdom

https://ilaebritish.org.uk/events/2022‐ilae‐british‐branch‐clinical‐epilepsy‐1‐day‐course/

### American Epilepsy Society Annual Meeting

2‐6 December 2022

Nashville, Tennessee

http://www.aesnet.org/AES‐annual‐meeting

### 48th Annual Meeting of the International Society for Pediatric Neurosurgery

6‐10 December 2022

Singapore

https://www.ispnmeeting.org/2022/

### 12th World Congress for Neurorehabilitation

14‐17 December 2022

Vienna, Austria & Virtual congress

https://www.wfnr‐congress.org/

## 2023

### 12th EPODES Advanced Course

16‐20 January 2023

Brno, Czech Republic

https://www.ilae.org/congresses/12th‐epodes‐advanced‐course

### Arbeitstagung NeuroIntensivMedizin (ANIM 2023)

19‐21 January 2023

Berlin, Germany

https://anim.de/

### International Congress on Structural Epilepsy & Symptomatic Seizures

29‐31 March 2023

Gothenburg, Sweden

http://www.stess2023.se/

### 15th European Pediatric Neurology Society Congress (EPNS): From Genome and Connectome to Cure

20‐24 June 2023

Prague, Czech Republic

https://www.epns.info/epns‐congress‐2023/

### 20th International Congress of Neuropathology

13‐16 September 2023

Berlin, German

https://www.ilae.org/congresses/20th‐international‐congress‐of‐neuropathology

### 10th Eilat International Educational Course: Pharmacological Treatment of Epilepsy

8‐13 October 2023

Jerusalem, Israel

https://www.eilatedu.com/

## 2024

### Seventeenth Eilat Conference on New Antiepileptic Drugs and Devices (EILAT XVlI)

5‐8 May 2024

Madrid, Spain

